# The Sustainability of Using Domestic Tourism as a Post-COVID-19 Recovery Strategy in a Distressed Destination

**DOI:** 10.1007/978-3-030-65785-7_46

**Published:** 2020-11-28

**Authors:** Erisher Woyo

**Affiliations:** 1 Department for Informatics, Technical University of Munich, Garching bei München, Bayern Germany; 2 Smart Tourism Education Platform (STEP) College of Hotel and Tourism Management, Kyung Hee University, Seoul, Korea (Republic of); 3 Department of Tourism and Service Management, MODUL University Vienna, Vienna, Wien Austria; Namibia Business School, University of Namibia, Windhoek, Namibia

**Keywords:** Distressed destination, Coronavirus, Tourism recovery, Domestic tourism, Resilience

## Abstract

Tourism is a critical contributor to the gross domestic product, especially among developing countries like Zimbabwe. Zimbabwe is a tourist destination that relies more on international travellers, a market which has been affected by the novel coronavirus. The purpose of this study is to establish the perceptions of domestic travellers and tourism managers on the sustainability of using domestic tourism as strategic responses to the impacts of the coronavirus. This study employs a qualitative methodology to examine the perceptions of the demand and supply side regarding the recovery options for Zimbabwean tourism post-pandemic. Online interviews with demand and supply participants were conducted. Data were analysed using thematic analysis, and the results were discussed. Results show that domestic tourism as a recovery option is unstainable due to the challenges that Zimbabwe is facing, beyond the coronavirus.

## Introduction

Coronavirus (COVID -19) is a global health pandemic that has triggered an unprecedented crisis in the tourism industry globally [1], making it the most powerful phenomenon of the 21^st^ century [2]. The pandemic saw the growth of the tourism industry revised downwards, and it is estimated that 75 million jobs have been lost following the suspension of international travel by March 2020 [3]. It is further estimated that the industry is likely to lose more than US$2.1 trillion in revenue [3] due to the closure of national borders and lockdowns. Therefore, the impacts of COVID-19 on destinations that overly rely on tourism are devastating.

Though COVID-19 is not yet contained, countries including Southern African countries are easing the lockdown through the opening of national borders to restart international travel. The easing of restrictions has been necessitated by business and political voices that are pushing for the opening of economies as soon as possible [4]. In anticipation of the resumption of travel, destinations developed tourism recovery strategies for an industry that contributes much to the gross domestic product (GDP) of most developing economies. Zimbabwe launched its tourism recovery strategy in August 2020 intending to promote domestic tourism as a strategic option and means of building destination resilience.

Domestic tourism promotion as a recovery strategy is not new in literature. It has been a default response in several destinations during crises in countries like Kenya, following post-election violence in 2008, and Malaysia during the Asian financial crisis1997/98 [5]. Though extant research has evaluated the vulnerability of destinations following natural disasters [6], research focusing on the promotion of domestic tourism as a destination recovery and resilience building strategy during pandemics in destinations with prolonged political and economic crises like Zimbabwe is limited. Understanding the sustainability of promoting domestic tourism as an alternative following the decline of international tourism demand due to the impacts of COVID-19 is required. Globally, due to COVID-19, the tourism industry is confronted with severe demand and supply challenges. These challenges vary from one country to another. Generally, the aspects of perceived health, social and psychological risks top demand challenges, while the supply side is confronted with challenges including deficits, low occupancies, job losses, company liquidation, and human capital depletion [1, 2, 7]. Based on this, the objective of this study is to examine the sustainability of using domestic tourism as a recovery and resilience building strategy using both demand and supply-side views in a destination with ongoing political and economic challenges.

## Literature Review

Tourism is a sensitive industry [8], especially to disasters and crises [9]. Due to the growing amount of disasters, literature discussing disasters is also growing [2]. It has been established that natural disasters do not result in permanent effects on tourism compared to the effects of incidents like violence and terrorism [10]. Disasters and crises are terms that are often used interchangeably in the literature, though fundamental differences exist [11–13]. On the one hand, a crisis is a disruptive event or outcome occurring within a system and has the potential to threaten the system’s [14]. Following the contested land reform programme, political and economic crises, have been a common occurrence in Zimbabwe [8]. These crises threatened the operations of tourism, resulting in dwindling arrivals and income [8]. On the other hand, a disaster is an event that confronts a system with unpredictable catastrophic changes over which a system has no control [13]. Thus, crises have an internal outlook that makes them more manageable, while disasters are external and less predictable [15].

It is not clear in literature if COVID-19 is a disaster or crisis. Zenker and Kock [2] argue that it is either a disaster or crisis, depending on the lenses one uses to analyse its impacts. While the classification of COVID-19 is contestable, it is beyond the scope of this study. However, its categorisation is critical to better our understanding of the past [2] and improve the formulation of tourism recovery and resilience strategies post-pandemic. Though COVID-19 is new, several of its aspects have been experienced and affected travel and tourism [16, 17]. COVID-19 in this study is treated as a unique disaster based on its scale and impacts on the global economy. COVID-19 is a natural disaster that degenerated into a socio-political, tourism and economic crisis [2].

The impacts of COVID-19 on the tourism industry globally are yet to be fully developed and measured [18]. However, what is clear is that following the closure of borders, the demand for international tourism plummeted [1]. The recovery of global tourism, regardless of the type of the crisis, depends mostly on the scale, type, and size of the crisis [15]. With no vaccine in sight, it appears that the pandemic will be around for a long-time, with some countries already been hit by a second wave. Therefore, the tourism recovery efforts must be formulated based on dealing with an ongoing pandemic. This is being reflected in how countries are opening their economies and national borders. Despite an increased stream of literature investigating disasters and crises in tourism, these studies are limited in terms of scope and depth [6, 19], and the promotion of domestic tourism as a means of recovery and resilience building, is hardly discussed. Furthermore, though the focus of most studies has been post-crisis/disasters, they have not dealt with a pandemic with socio-economic impacts that are so devastating like COVID-19. Thus, the understanding of the role that domestic tourism promotion plays in helping destinations with ongoing crises and negative reputations reduce vulnerability and building resilience is critical.

Debate on what constitutes resilience is ongoing [20], suggesting that it is another elusive term in tourism research. Resilience is an essential construct in disaster and crises management research because it provides destination managers with the means of enhancing capacity to adapt and deal with changes [21, 22]. Though there is a lack of universal measurement, resilience has been approached using three significant perspectives: engineering, ecological and adaptive perspectives [23–25]. Resilience from an engineering perspective measures how rapid a system can return to its normal state. In contrast, the ecological view measures resilience using the system’s ability to absorb the impacts of disruption without altering its identity, functions, and structure [25]. Adaptive resilience refers to the systems’ ability to experience the impacts of changes without losing the ability to manage its resources. Thus, in a destination context, resilience is concerned with how quick the destination can return to its previous normal conditions following a shock [25], such as COVID-19. Adaptive destinations can adjust to, learn from, and manage changes [26]. Applying adaptive resilience thinking during disasters and crises provides “great utility, namely, the ability to react to external stimuli and modify behaviour accordingly” [27 pp. 4]. COVID-19 presents an opportunity for the promotion of domestic tourism to destinations such as Zimbabwe that relies heavily on international tourism. Whether this strategy could prove to be useful to a destination with ongoing crises [8] remains relatively unknown. Thus, the recognition by destinations of vulnerabilities and being able to plan for eventualities is critical tourism recovery and building a resilient destination. Destinations that can adapt are thus considered more resilient and can recover from disasters and crises quicker [28].

The capacity of a tourist destination to respond to pandemics is framed in stages [4]. Despite knowing that tourism is a victim of crises and disasters [27], the conceptualisation of recovery and resilience building strategies is generally informed from a reactive point. COVID-19 has long-term impacts on both international and domestic tourism. Thus, how destinations with ongoing political and economic crises respond to the pandemic in shaping long-term tourism recovery and resilience building strategies depends on how well-thought and planned the strategies are. Following the decline in international tourism demand, spearheading tourism recovery and enhancing the resilience of the sector through the growth and promotion of domestic tourism is timely and critical, especially for destinations that overly relies on international visitors.

## Methodology

The study examines the perceptions of demand and supply participants regarding the promotion of domestic tourism as a post-pandemic recovery and resilience building strategy in Zimbabwe. Zimbabwe is a Southern African tourist destination with several world-class attractions including the Victoria Falls, Hwange National Park, Gonarezhou National, Eastern Highlands and Great Zimbabwe Monuments [29]. International tourism, which is the biggest market to Zimbabwe, has shown some resilience after it was disrupted in 2000 due to the contested elections, violence, and the land reform programme [8, 29]. These pre-COVID crises in Zimbabwe are not new in literature. However, COVID-19 presents a situation which is novel and not much is known regarding this ongoing virus. Based on this, Strauss and Corbin [30] argue that a qualitative methodology is appropriate in cases where newer problems for which nuanced understanding needs to be developed. Thus, a qualitative research approach was employed.

Data were collected using online interviews (in English) between May and July 2020. Due to COVID-19 restrictions, online interviews are becoming a standard method of data collection. Online interviews were conducted using Zoom Video Conferencing Platform with both the demand side (domestic tourists) and supply-side (managers from tourism establishments) participants. These interviews were conducted separately. Questions used in the interviews were based on the review of the literature. Demand interviews focused on the perceptions and challenges faced by domestic travellers in Zimbabwe. Supply side interviews focused on the feasibility of using domestic tourism as a means of restarting tourism, challenges of using domestic tourism and adoption of technologies.

Thirty demand respondents were approached using WhatsApp messages, where the aims of the study were explained. Twenty participants accepted to participate (66.7% response rate). Demand participants were supposed to have visited major attractions such as the Victoria Falls, Great Zimbabwe and big national parks including Hwange and Gonarezhou in the last three years to participate in the study. 28 managers were initially approached using emails, and 12 accepted to participate in the study (42.8% response rate). Both demand and supply participants were sampled using convenience sampling. Occasional email and WhatsApp reminders were sent to participants. Participants were informed of the content, aims, their rights to anonymity, and withdrawal of participation. Interviews were recorded using the Zoom cloud facility, averaging 20–30 min. Tables 1 and 2 summarises the participants’ profiles.

Interviews were conducted, transcribed and analysed using thematic analysis. Data analysis started full data transcription, followed by data familiarisation, codes identification, searching, reviewing, and defining themes, and generation of results. Coding was performed manually, through repeated reading of and making notes on interview transcripts.

**Table 1.** Supply side profile

Participant	Position	Establishment	Age		Gender	Experience	Location
S1	General manager	5-star hotel	40		Male	15 years	Harare
S2	General manager	4-star hotel	39		Male	12 years	Victoria Falls
S3	General manager	Tour operator	36		Male	8 years	Victoria Falls
S4	General manager	Restaurant	41		Male	6 years	Victoria Falls
S5	General manager	Tour Operator	50		Male	14 years	Victoria Falls
S6	General Manager	Museum	34		Female	5 years	Master
S7	General manager	Tour Operator	33		Female	5 years	Harare
S8	General manager	3-star hotel	38		Female	5 years	Masvingo
S9	General manager	3-star hotel	36		Female	5 years	Nyanga
S10	General manager	Car rental	30		Female	5 years	Kariba
S11	General manager	Tour Operator	37		Female	13 years	Kariba
S12	General manager	Restaurant	42		Female	10 years	Harare

**Table 2.** Demand-side profile

Participant	Age (years)	Gender	Income (ZWL)*	Qualification	Residing
D1	27	Male	$3 200	Bachelor’s	Urban
D2	50	Female	$1 000	Diploma	Urban
D3	44	Male	$5 000	Bachelor’s	Urban
D4	35	Female	$20 000	Higher diploma	Urban
D5	59	Male	$15 000	Certificate	Peri-urban
D6	34	Female	$5 500	Bachelor’s	Urban
D7	39	Female	$7 000	Secondary	Urban
D8	31	Male	$15 000	Bachelor’s	Peri-urban
D9	35	Male	$12 000	Bachelor’s	Urban
D10	38	Male	$18 000	Bachelor’s	Urban
D11	48	Female	$15 000	Postgraduate	Farm
D12	42	Male	$30 000	Master’s	Rural area
D13	27	Female	$20 000	Master’s	Urban
D14	28	Female	$8 000	Master’s	Urban
D15	36	Male	$10 000	Doctorate	Urban
D16	49	Male	$1 000	Bachelor’s	Urban
D17	32	Female	$9 000	Bachelor’s	Urban
D18	29	Female	$8 500	Diploma	Urban
D19	27	Male	$25 000	Bachelor’s	Urban
D20	52	Male	$20 000	Master’s	Urban

^*^At the time of writing, USD$1 = ZWL$50

## Findings

This section discusses the sustainability of using domestic tourism as a recovery and resilience building strategy. The key issues that emerged from the analysis of data are discussed in the following sections.

### Affordability of Tourism Products and Services

The theme of affordability of tourism products revealed deep-seated challenges that Zimbabwean destination managers need to deal with if the promotion of domestic tourism is to pay off. Using demand analysis, tourism in Zimbabwe is perceived as expensive. Results show that many domestic tourists cannot afford to travel for leisure in Zimbabwe. Thus, demand participants unanimously agree that affordability of tourism products is a critical concern that should be addressed if Zimbabwe wants to pin its tourism recovery using the domestic market as noted below:

- *D7: “The elite may do that, but I am not sure if they are also ready to support the local industry, as they can visit other destinations. Yes, domestic tourism could work, it might take off, but at an incredibly low pace because we cannot afford their prices now.”*
- *D16: “How could I travel, when I am earning less than USD$30, after working for the whole month.*

Cost of travel was identified as an inhibitor of domestic travel, thus affecting the success of using domestic tourism as a recovery and resilience building strategy in Zimbabwe. While there was a particular cost-related question in the interview guide, the aspect of the cost of travel as an inhibitor of domestic travel emerged in the answers long before the question was asked and it is illustrated as follows:

- *D8: “Tourism has a positive relationship with the availability of disposable income. To then say domestic tourism could be a recovery strategy is problematic because if that positive relationship is looked at, it means Zimbabwe must recover first for disposable income to be available.*
- *D4: “Imagine a civil servant who earns less than US$30 and is expected to pay US$150 per night in a decent hotel, what is that prices should be lowered like what South Africa charges”.*
- *D15: “Look at bungee jumping, it attracts a charge of US$120 per jump, rafting US$115, and a helicopter flight US$150 for 12 min. There should be prices tailored for locals so that we also enjoy these attractions”.*

The aspect of inclusion remained a recurring sub-theme in most demand responses:

- *D2: “Let us begin with financial inclusion; then affordability for local tourists. We love travelling every holiday we go to kumusha/ekhaya [to the countryside]. We could visit Vic falls or Nyanga if the trips do not burn holes in our pockets.*
- *D9: “Tourism in Zimbabwe was not designed for us domestic clients; it was designed for varungu [international visitors].*

### The Willingness of the Domestic Market to Pay for Tourism

Willingness to pay in this analysis developed as a factor explaining how the supply participants perceive the viability of recovering tourism and building a resilient destination using domestic tourism in Zimbabwe in the absence of international tourism due to COVID-19. Many supply-side participants note that domestic tourism might not be sustainable, given their unwillingness to pay premium prices:

- *S8: “The domestic market has many challenges, and key among them is lack of disposable income to support travelling and paying the premium prices we often charge.”*
- *S9: “We are pricing ourselves out of business. I think to entice domestic tourists we should work on the law of numbers. There is more to benefit by charging $20 per night and get lots of people regularly coming than charging $100 and get four customers per night occasionally. I do not know the economics we are using. The food is also expensive.”*

Moreover, some supply participants’ reflections of kickstarting using domestic shows that post-pandemic, it will not deliver desired results, given that its promotion following post-2000 crises has been futile:

- *S6: “Zimbabwe has always been struggling to stimulate domestic tourism, will it do that after the pandemic, I doubt”.*
- *S9: “Numbers are too little. Of the many millions in Zimbabwe, very few are mobile and resourced (the elite)”*
- *S10: “This is not the first time we hear the use of domestic tourism, the last two-three years, ZTA has been making noise about this, but with little changes. Even when Zimbabwe said they were looking East, changes in numbers have not been that large, more effort is required”.*
- *S11: “Most Zimbabweans are low-income earners that cannot economically support tourism in Zimbabwe. Furthermore, the economic climate, compounded with COVID-19, makes it significantly worse, and I do not see domestic tourism supporting the revival of the sector, maybe I am too sceptical”.*

Although the majority supply participants were sceptical, some participants argue that post-pandemic tourism recovery and post-pandemic resilience needs to go beyond the promotion of domestic tourism:

- *S6: “It is short-term opium. It is too dependent on disposable income.”*
- *S7: “It will help with hotel occupancies here and there, but the value is too low for what this property can go for”.*
- *S10: “As a tourism-dependent country, it may work, but in the long-run, we will not be able to compensate for at least some of the lost income with domestic tourists.*
- *S12: “Domestic tourists take short-term trips, generally, about 1 to 3 days, and this will not be meaningful in short to long term in terms of occupancies”.*

### Economic Performance

Zimbabwe is faced with an ongoing economic crisis that stemmed from high inflation, unemployment, drought, and a worsening political climate. Despite that, pre-COVID-19 has been a significant contributor to the country’s GDP [8, 29]. The performance of the economy was described by supply as the primary deterrent of using domestic tourism, given the lack of economic fundamentals:

- *S5: “Even with international tourism pre-COVID, we still have supply challenges that are attributed to a poor performing economy, including limited access, and lack of airline connectivity. The international market has been resilient, but not sure how it will work out for the domestic market.”*
- *S8: “Too much unemployment and high inflation, makes the tourism recovery using domestic tourism largely too ambitious”.*

Demand views concerning how the performance of the economy affects the use of domestic tourism as a recovery and resilience option were mainly from the fact that many are unemployed, and earnings are meaningless:

- *D7: “Hatina mabasa (we have no jobs), how then can we travel in such an economy”.*
- *D20: “Hatitambiri baba (we are not earning), so there will not be any leisure to make”.*

### Adoption Technologies

Because of social distancing, the study also asked supply participants if Zimbabwean tourism could adopt information and communication technologies (ICT) including virtual reality (VR). Adoption of technology in this study is established as a theme that explains supply views in circumstances where tourists are unable to fly. Mixed opinions are noted regarding the adoption of ICT in tourism in a distressed destination:

- *S1: “The use of ICT in the tourism industry is limited but could work if the government creates an enabling environment and stop using the internet as a means of power. Shutting down the internet is bad for business, should we start selling virtual tourism”.*
- *S3: “Virtual maybe somewhere. In Zimbabwe, we are even failing to have virtual meetings. It is embarrassing. Forget about virtual tourism as a strategic response to the impacts of COVID-19”.*
- *S5: “Zimbabwe’s ICT infrastructure needs a facelift, and then we can talk about virtual tourism. With internet shutdowns that are always instigated by the authoritarian regime we have now; this might not work”.*
- *S9: “Imagine, selling a virtual reality experience, where people across the world can have an experience of Victoria Falls or Great Zimbabwe through a simulated environment? This can potentially revolutionise the tourism industry, especially now that most people across the world are confined to their homes, in the spirit of maintaining social distance. But then, look, we have much more deep-seated challenges, that might not support the use of ICTs in tourism in Zimbabwe”.*

## Discussion and Conclusion

**Fig. 1.**
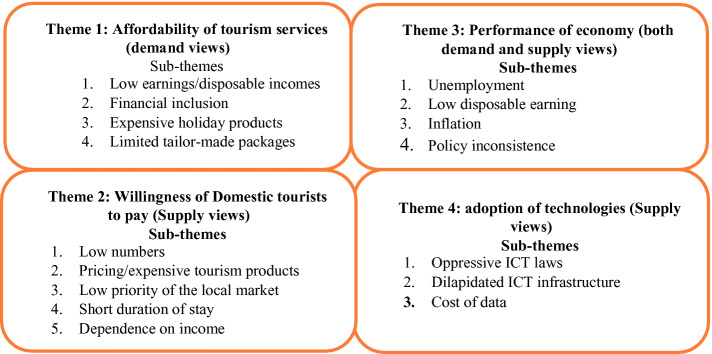
Factors affecting domestic tourism as a recovery and resilience strategy in Zimbabwe

This study examines the perceptions of demand and supply participants concerning the use of domestic tourism as a post-pandemic recovery and resilience strategy. The impacts of COVID-19 on tourism are likely to remain for an extended period in the absence of a vaccine, and the sector must be more prepared than it was when the pandemic struck. Post-COVID tourism recovery is likely to be slow and will depend on the recovery of the global economy as well as the risk perceptions of travellers in the absence of pharmaceutical solutions. The recovery of Zimbabwean tourism using domestic tourism is likely to be slowed by four key factors. These factors include the affordability of the tourism product by the domestic market, willingness of domestic tourists to pay, the performance of the economy and adoption of technologies. Each of these themes had several different sub-themes, as presented in Fig. 1.

The following main findings were evident from the analysis of data. Firstly, Zimbabwean tourism is expensive for the market that should be leading the post-pandemic tourism recovery and the creation of a resilient destination. It is imperative to highlight that the viability of using domestic tourism as an option for recovery and resilience building of the tourism industry depends on how attractive and affordable the tourism product is. Several demand participants argued that pricing of the tourism product makes it difficult for them to participate in and consume tourism activities. Therefore, destination managers must consider price reductions and other incentives in stimulating the domestic market. Given the negative relationship between the price of tourism products and demand for tourism, recovery and resilience building effort must ensure the aspects of affordability is addressed.

Both supply and demand participants agreed that domestic tourism is viewed as something which is beyond the affordability of the domestic market. This challenge has also been identified in studies that involved international tourists, where it was concluded that Zimbabwe is an expensive destination compared to regional competition [8, 29]. Results from demand and supply views show the need for a pricing regulatory framework in Zimbabwe aimed at boosting domestic arrivals in the interim and international arrivals in the long-term. This could help build destination loyalty, which was noted as weak in past studies [8, 29]. The price regulatory framework will also encourage both domestic and international tourists to stay longer than the reported average of 1–2 days at a destination [29]. Failure to address this will result in the destination losing more revenue and market share. This will add to the existing distress in terms of unemployment to the destination.

Secondly, the findings show the immediate need for inclusively promoting tourism. Domestic tourists feel that they are excluded in the tourism value chain due to exorbitant prices and the packages that are offered by the supply side. Some supply side participants also indicated that much priority, even in terms of marketing, has always been given to the international market. While efforts are being made to market domestic tourism aggressively, there is a need to develop packages that are tailor-made for the domestic market. Competitive prices must be deliberately pursued as a means of building financial inclusion, and government can play a crucial role in making subsidies available for the sector to charge less for domestic travellers. The pricing policy of Zimbabwe, which favours low volume and high spending visitors should be revised given the changing market dynamics, even for international tourism [8, 29]. Thus, addressing price competitiveness issues for a destination in distress. It is relatively challenging to promote domestic tourism as a recovery and resilience building strategy post-pandemic in a country where citizens have low disposable income, and the services are expensive (see Table 2).

Thirdly, the supply side is sceptical regarding the contribution of domestic tourism in enabling tourism recovery and resilience in Zimbabwe. Zimbabwe’s pre-COVID-19 economic challenges which have been exacerbated by the pandemic does not promote tourism, especially from the domestic front. Tourism is highly dependent on income, and in the context of Zimbabwe, the domestic tourism market is highly income constrained. While it is recommended in the literature that at home is safer than visiting abroad during crises and disasters [10, 31], the findings of this study seem to suggest otherwise. Though supply participants lauded the use of domestic tourism as a viable strategy, they were quick to hint that it is a short-term strategy that might not help Zimbabwe to recovery and be a resilient destination given its over-reliance on international travellers [29]. As earlier indicated, there is a need also to market the domestic tourism aggressively given the low priority the destination management organisation has accorded it. Additionally, this aggressive marketing must be supported by ensuring government and insurance schemes provide access to health care away from home. In the context of Zimbabwe, the assurance of insurance schemes and provision of health care away from might be difficult given its underdeveloped health system and a collapsing economy. There is a need for destination managers to increase the recovery rate by promoting destinations to international travellers through a range of tactics including enhancing the image of air travel safety in the long-term.

Fourthly, the performance of the Zimbabwean economy remained a recurring response in this study. Macro-economic factors will determine tourism recovery post-pandemic, and these fundamentals are not right in Zimbabwe, given the issues of hyperinflation, unemployment, corruption, and rising debt. Supply results showed that Zimbabwe has no capacity for funding the promotion of domestic tourism. Aggressive promotion of domestic tourism as a recovery and resilience strategy requires funding from the government. As part of their tourism recovery efforts, the Swiss and New Zealand governments, for instance, committed USD$42.2 million and USD$256.8 million to fund the promotion of domestic tourism. Currently, there is no known amount committed by the Zimbabwean government towards the promotion of domestic tourism as a recovery and resilience strategy post-pandemic. The success of using domestic tourism as a recovery strategy requires substantial funding in terms of subsidies and incentives. However, the strategy might not bring in results given the pre-COVID-19 current economic challenges that Zimbabwe has been experiencing. Thus, the use of domestic tourism strategy is merely an academic and theoretical one in the empirical context of Zimbabwe. Incentives like price reductions, tax incentives and subsidies are imperative given that domestic tourism is a market that is price sensitive. Subsidies and other forms of cash injections are imperative in keeping the industry functioning; otherwise, unemployment is likely to increase, and further affecting recovery efforts and puts Zimbabwe in deepening economic distress. This will also make it difficult for establishments to charge lower prices in the absence of incentives and subsidies, give higher operational costs in the sector.

Domestic tourism is perceived to recover quicker to pre-COVID-19 levels compared to international travel in other contexts. However, for this to happen, there is a need to increase accessibility to domestic attractions. Previous research argued that Zimbabwe is relatively inaccessible due to lack of internal airline connections, heavy police presence on the highways, and bad roads [8, 29]. Therefore, the practical promotion of domestic tourism requires government and other role players to address these supply-side challenges. Lastly, the adoption of technology, in particular robots and artificial intelligence, has been argued for in literature [32, 33]. In the view of the scepticism expressed by supply participants regarding the use of domestic tourism as a means of restoring tourism and building resilience, the possibility of implementing technologies was also explored. Though technology adoption could benefit Zimbabwean tourism, the economy and political players remains critical deterrents. The implementation of robots and other types of technology in travel and tourism needs an enabling infrastructure, which Zimbabwe does not seem to provide. Zimbabwe is one of the countries with oppressive ICT laws and in the past, the internet was permanently shut down [34], despite the new president promising a different narrative than what was experienced during the Mugabe administration [8]. Stakeholders must come together and create applications that stimulate the experience of visiting Zimbabwe and package them for sale to potential clients around the world.

This study contributes to literature in the form of different themes than can be regarded as useful factors in further research. The dominant themes of affordability, the willingness of the domestic market to pay, the performance of the economy and adoption of technologies should be taken as an imperative contribution to theory regarding tourism recovery and resilience during the COVID-19 pandemic. However, this study is not without limitations. The study was based on a qualitative approach using a conveniently selected sample which may not justify generalisability of the results. Rigorous empirical investigations regarding the promotion of domestic tourism as a recovery and resilience strategy must be conducted. Future studies must also review pre-COVID and post-COVID challenges in promoting domestic tourism as this will provide the destination with an analytical framework that could help develop sustainable recovery and resilience building strategies of the industry. Though several aspects of COVID-19 have been reported in past studies, there is a need for research to provide a more nuanced understanding of the industry responses to a pandemic with wide-ranging impacts, including scale development. The findings of this study are context-specific; thus, themes and challenges argued in this study may not apply in other destinations, even if they have ongoing crises like Zimbabwe.
